# RNA Vaccines: A Suitable Platform for Tackling Emerging Pandemics?

**DOI:** 10.3389/fimmu.2020.608460

**Published:** 2020-12-22

**Authors:** Jonas B. Sandbrink, Robin J. Shattock

**Affiliations:** ^1^Medical School, Medical Sciences Division, University of Oxford, Oxford, United Kingdom; ^2^Department of Infectious Diseases, Imperial College London, London, United Kingdom

**Keywords:** mRNA vaccine, infectious disease, pandemics, outbreak, vaccine development, vaccine platform, self-amplifying RNA

## Abstract

The COVID-19 pandemic demonstrates the ongoing threat of pandemics caused by novel, previously unrecognized, or mutated pathogens with high transmissibility. Currently, vaccine development is too slow for vaccines to be used in the control of emerging pandemics. RNA-based vaccines might be suitable to meet this challenge. The use of an RNA-based delivery mechanism promises fast vaccine development, clinical approval, and production. The simplicity of *in vitro* transcription of mRNA suggests potential for fast, scalable, and low-cost manufacture. RNA vaccines are safe in theory and have shown acceptable tolerability in first clinical trials. Immunogenicity of SARS-CoV-2 mRNA vaccines in phase 1 trials looks promising, however induction of cellular immunity needs to be confirmed and optimized. Further optimization of RNA vaccine modification and formulation to this end is needed, which may also enable single injection regimens to be achievable. Self-amplifying RNA vaccines, which show high immunogenicity at low doses, might help to improve potency while keeping manufacturing costs low and speed high. With theoretical properties of RNA vaccines looking promising, their clinical efficacy is the key remaining question with regard to their suitability for tackling emerging pandemics. This question might be answered by ongoing efficacy trials of SARS-CoV-2 mRNA vaccines.

## Introduction

The coronavirus COVID-19 pandemic demonstrates the threat posed by pandemic pathogens. Emerging pandemics are epidemics with potential to spread worldwide caused by potentially novel or emerging pathogens able to rapidly spread in the absence of pre-existing protective immunity. Sociological and ecological factors have favored zoonotic emergence of such pandemics, while biotechnological advances may have increased the danger from human-originated pandemics (1, 2). Indeed, advances in gene synthesis and editing technology may enable actors with malicious intent to synthesize mutated, novel, or previously eradicated pathogens for deliberate release. Vaccines are a powerful tool to counter infectious diseases threats with pandemic potential. However, vaccine development from preclinical phase to licensing takes on average more than 10 years (3). Hence, currently, vaccine development is too slow for vaccines to be available to control an emerging pandemic caused by Disease X.

A growing number of agencies have been engaged in pandemic surveillance and preparedness including agencies such as the WHO, Gavi, BARDA, CDC, and the risk of infectious threats remains high on many national agendas (2). To tackle this issue, the Coalition for Epidemic Preparedness Innovations (CEPI) has set the goal for new vaccines to reach clinical testing within 16 weeks of pathogen detection and for 100,000 doses to be produced within 30 weeks (4). To meet these goals, CEPI has called for the advancement of platform vaccine technologies. This refers to vaccine systems where a universal backbone can be adapted to target different pathogens. RNA vaccines are one such platform which promises fast vaccine development, approval, and manufacturing. Hence, CEPI has funded the development of mRNA vaccines, including the development of SARS-CoV-2 vaccines, by the biotech companies CureVac and Moderna, as well as the development of self-amplifying mRNA (saRNA) vaccine technology by academics at Imperial College London (5–7).

Speed of vaccine development, approval, and manufacturing is not the only characteristic necessary for a vaccine to be suitable for tackling an emerging pandemic. For global availability of a vaccine during a pandemic, scalable and low-cost manufacturing and vaccine thermostability are needed. A given platform should be able to induce both cellular and humoral immunity to be able to tackle novel pathogens in absence of an established correlate of protection, ideally with a single dose. Most importantly, safety, tolerability, and efficacy of a given vaccine need to be shown. This article will evaluate in what ways RNA-based vaccines meet these criteria to date and what steps need to be taken to improve the suitability of RNA vaccines for a pandemic setting. A focus will be laid on vaccines against RNA viruses with particular pandemic potential, such as influenza virus and coronavirus (8). Findings from vaccines targeting other pathogens which provide useful insights into the properties and current state of the RNA vaccine platform will also be considered.

**Figure 1:**
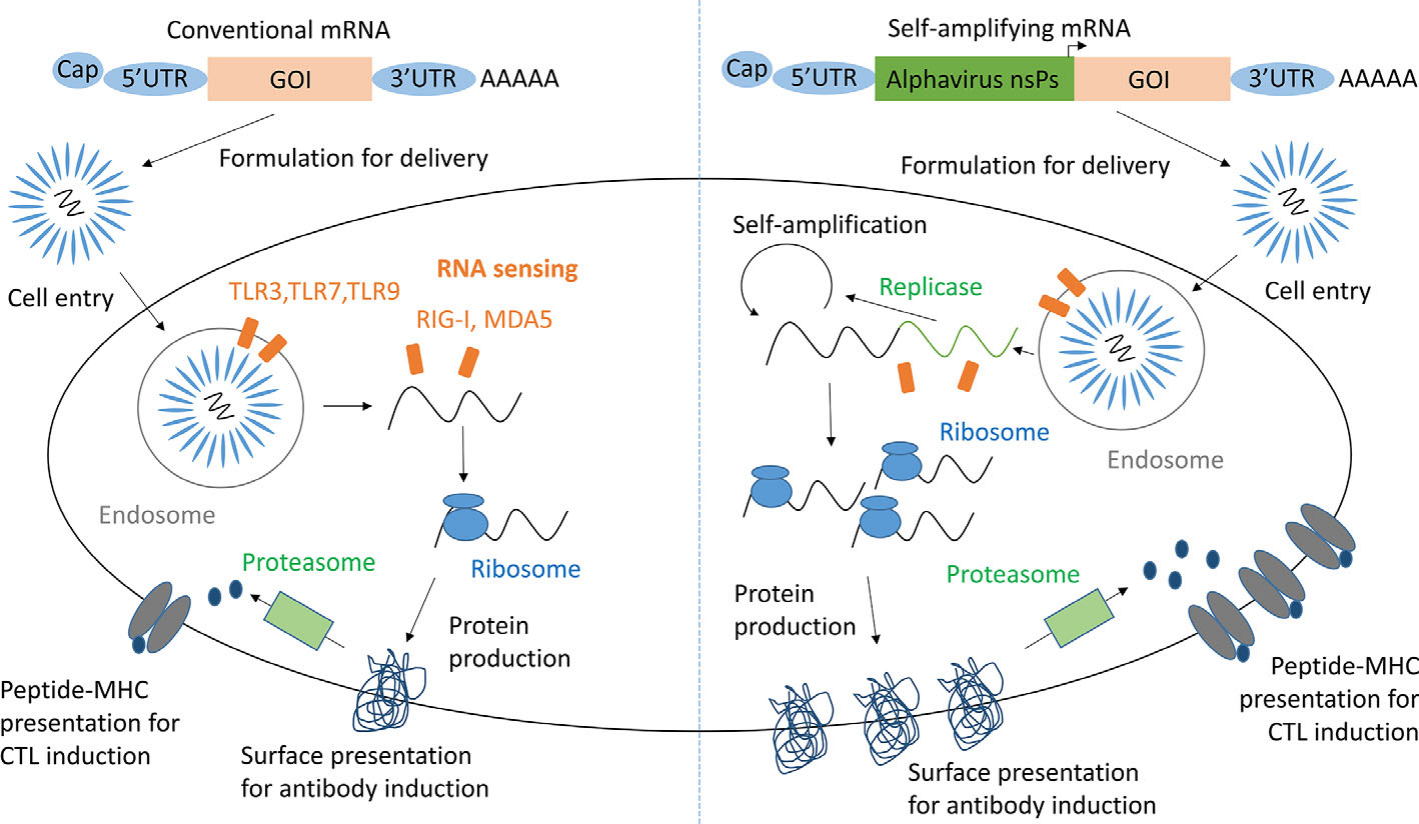
mRNA and saRNA protein production in antigen presenting cells. Adapted from Maruggi et al. (9). GOI, Gene of interest; UTR, Untranslated regions; nsPs, non-structural proteins; CTL, cytotoxic T lymphocyte.

RNA vaccines are based on the premise that mRNA, injected for vaccination, when taken up by antigen presenting cells (APCs) and other target cells induces expression of the properly folded and glycosylated antigenic protein (**Figure 1**) (10). As RNA activates endosomal and cytosolic RNA sensors upon cell entry, these vaccines exhibit self-adjuvanticity and induce both a humoral and cellular immune response against the encoded protein (11, 12). As saRNA vaccines contain a replicon based on alphavirus non-structural proteins, they are able to self-amplify within host cells (13). Hence, saRNA vaccines have the potential to induce higher levels of protein production and immunogenicity relative to the injected dose compared to conventional mRNA vaccines (14).

Self-adjuvanticity of RNA may be a double-edged sword. While enabling self-adjuvancy, recognition of foreign RNA by intracellular RNA sensors may also induce its degradation and/or silencing of expression (15). RNA modification strategies such as: the addition of a 5’ cap; the length and structure of a 3’ PolyA tail; use of untranslated regions and the modification of nucleosides, and sequence optimization, have been critical for decreasing the degradation and increasing the immunogenicity of mRNA (16–18). Furthermore, RNA sequence optimization may raise expression efficiency and hence immunogenicity (19, 20). Additionally, RNA may be formulated with a delivery vehicle, such as lipid nanoparticles (LNPs) or cationic polyplexes, which protect RNA from degradation, boost target cell uptake, and increase adjuvancy (21).

## RNA Vaccines Promise Fast Development and Scalable Manufacturing

RNA vaccines promise fast development as upon sequencing of the target pathogen mRNA candidates can be designed and synthesized very quickly. Additionally, reduced need for optimization and regulatory testing of a new vaccine may further speed up development and the approval process (22). This is because a new RNA vaccine will only differ in the encoded sequence of its target protein, while its formulation and method of delivery will have been optimized and licensed in the past. Indeed, Moderna made news by starting human testing of a SARS-CoV-2 mRNA vaccine only 66 days after viral genome sequencing (23). This unparalleled feat was not only enabled by sequence-informed design and fast manufacturing of the mRNA vaccine for clinical testing, but also by previous work on an mRNA vaccine for MERS-CoV, which informed antigen selection and prefusion stabilization of the SARS-CoV-2 spike protein (23). This supports the notion that in addition to technical advancement of fast-response vaccine platforms applying these platforms to prototype pathogens within viral families of high pandemic potential might be critical for pandemic preparedness (24).

RNA vaccines also have promising properties with regard to manufacturing. Due to similarity between vaccines, a single set of production facilities may be used and production may be swiftly switched to that of a new vaccine for tackling an ongoing outbreak. mRNA is produced by *in vitro* transcription from a linear DNA template by a bacteriophage RNA polymerase (12). Although current approaches predominantly use DNA templates generated from plasmid DNA produced in bacteria, even this step is likely to be replaced with fully synthetic approaches for generating linear DNA (25). This comparatively simple, high-yield, and virus-free production process enables production facilities to be small and scalable (26). Cell-free production means there is less concern with respect to adventitious agents, microorganisms unintentionally introduced into manufacturing, although similar requirements are needed to ensure endotoxin removal, sterility, and control of any residual reagents. Kis and colleagues have estimated that 1L of saRNA, sufficient for 575,000 doses, could be manufactured in 60h at around $0.30 per dose (27). However, additional cost consideration should consider formulation and distribution costs. Nevertheless, the RNA vaccine platform promises not only fast development but also scalable and low-cost manufacturing.

## Safety and Tolerability of RNA Vaccines Look Promising if Disease Enhancement Can Be Prevented

Safety and tolerability need to be proven for the licensing of any given vaccine. RNA vaccines are comparatively safe. There is no risk of pathogen reactivation and RNA is degraded *in vivo* with no risk of antigen persistence or integration into the genome (28). Self-replicating RNA could pose additional safety concerns when encoding fusion competent viral glycoproteins, however such theoretical concerns can be mitigated through the use of stabilizing mutations ensuring fusion incompetent structures (29). Although no anti-vector effects have been observed with saRNA, T cell recognition of the replicon proteins necessary for RNA amplification might limit the reusability of a given saRNA-based platform.

As the number of clinical studies of RNA vaccines against infectious diseases is limited, the tolerability data from phase 1 clinical trials to date looks acceptable (30–32). However, the cohort size of clinical trials to date has been small, leaving the potential for rare but potentially severe side effects.

One safety concern unspecific to RNA vaccines might be disease enhancement induced by vaccination. For example, the clinical trial of a formalin-inactivated vaccine against RSV in 1967 led to increased mortality in children upon viral infection caused by a type 2 helper CD4^+^ T cell (Th2) mediated eosinophil invasion of the lungs (33). Lung immunopathology after vaccination has been observed in animal models vaccinated with a range of SARS-CoV and MERS-CoV vaccines (34–36). Hence, concern for disease enhancement might also slow the development of SARS-CoV-2 vaccines. To reduce the risk of lung immunopathology, possible RNA vaccine candidates need to show that they induce a non-Th2 biased immune response. Geall et al. demonstrated in 2012 that an RSV saRNA-LNP vaccine induced twice as high Th1-associated IgG2a titers as Th2-associated IgG1 titers in rats (37). Histological analysis of lung tissue and comparison to a positive and negative control of vaccine-induced RSV exacerbation might have provided additional insight into the safety of this vaccine candidate. In phase 1 clinical studies, mRNA-based SARS-CoV-2 induced Th1-biased immune responses suggesting their safety with regard to disease enhancement (31, 32). Hence, RNA vaccine candidates may be suitable for use against diseases where vaccine-induced disease enhancement is a concern. It is not yet discernable whether RNA-based vaccine approaches universally induce Th1-biased responses and to what degree this depends on the encoded antigen. While classically Th1-polarized responses have been associated with induction of potent cell-mediated immune responses against intracellular pathogens and Th2-polarized responses with humoral immunity against helminthic pathogens, this Th1/Th2 dichotomy has been challenged through the discovery of other CD4^+^ T cell subsets (38). Almost certainly, different viral pathogens will require the induction of immune responses with different balances of Th1 versus Th2 CD4^+^ T cell activation.

The fact that no viral culture is required for vaccine development and manufacturing means there are few biosafety concerns around work on RNA vaccines and lack of need for viral modification promises a good biosecurity profile.

Overall, safety and tolerability of RNA vaccines looks promising but needs to be confirmed in larger scale clinical trials. RNA vaccines have the potential to be developed in cases where disease enhancement is a concern; still, thorough testing in these cases will be necessary and might delay vaccine development.

## Efficacy Needs to Be Demonstrated in Clinical Trials

With safety and tolerability looking promising, the key question for whether RNA vaccines will find use in pandemics is whether they will show immunogenicity and efficacy in human trials. Ideally, disease protection after a single dose is needed to ensure fast distribution of vaccines. Additionally, a given platform should be able to induce both cellular and humoral immunity, so generated vaccines are able to induce immunity against a wide range of viral pathogens with different properties and pathology.

Pre-COVID-19, a few human immunogenicity trials using known correlates of protection for evaluation of clinical efficacy had been published, overall showing moderate success (**Table 1**). In a Moderna H7N9 influenza vaccine trial, the tested mRNA vaccine induced haemagglutinin inhibition assay (HAI) titers of 1 > 1:40 in 96.3% of individuals and virus neutralizing (VN) titers of 1 > 1:20 in 100% of individuals (30). HAI > 1:40 is universally accepted as a correlate of protection for reducing risk of influenza infection by 50%. However, this correlate of protection is largely based on studies of seasonal influenza and has to our knowledge not been confirmed for H7N9 or other potential pandemic subtypes. Additionally, different authors have suggested VN titers between 1:20 and 1:80 as a correlate of protection, complicating the interpretation of the measured VN titers (40). None of the pre-COVID-19 tested rabies or influenza vaccines induced cellular immunity (**Table 1**).

**Table 1:** Published immunogenicity studies in humans to date.

Study	Type	Pathogen	Best-performing protocol	Immunogenicity
Alberer et al. (39)	mRNA	Rabies virus	80 µg, i.d. with needle-free system, three doses	22/27 had virus neutralizing titers of > 0.5 IU/ml. No T cell response detected.
Feldman et al. (30)	mRNA	Influenza H10N8	100 µg, i.m., two doses	23/23 had titers of HAI > 1:40, 20/23 had titers of MN > 1:20. No T cell response detected.
Feldman et al. (30)	mRNA	Influenza H7N9	25 µg, i.m., two doses	29/30 had titers of HAI > 1:40, 30/30 had titers of MN > 1:20. No T cell response detected.
Jackson et al. (31)	mRNA	SARS-CoV-2	100 µg, i.m., two doses	15/15 had PRNT80 titers above mean of convalescent serum. Moderate CD4^+^ and small CD8^+^ T cell response detected.
Sahin et al. (32)	mRNA	SARS-CoV-2	30 µg, i.m., two doses	10/10 had VNT_50_ titers above mean of convalescent serum. Moderate CD4^+^ and CD8^+^ T cell response detected.

HAI, hemagglutinin anhibition assay; MN, micro neutralization assay; PRNT80, plaque-reduction neutralization assay (measuring titers that reduce infectivity by 80% or more); VNT_50_, Virus neutralizing titers (measuring titers which neutralize 50% of virus or more).

Evaluating the recently published immunogenicity data of SARS-CoV-2 vaccines is more difficult in absence of a well-established correlate of protection for COVID-19. Phase I clinical data show that Moderna’s mRNA-1273 vaccine was able to induce neutralizing antibody titers similar to those found in convalescent serum samples after two injections (**Table 1**) (31). The fact that despite rapidly dropping antibody titers in convalescent individuals COVID-19 reinfection is uncommon and cross-reactive T cell responses between coronaviruses seem to confer some degree of protection indicates an important role of cellular immunity in preventing and controlling SARS-CoV-2 infection (41, 42). Hence, the ability of COVID-19 vaccines to induce cellular immune responses might prove critical for vaccine efficacy. While after vaccination with mRNA-1273 a moderate Th1-biased CD4 T cell response was measured, only minimal induction of CD8^+^ T cell cellular immunity was observed. Pfizer/BioNTech’s vaccine candidate BNT162b1, a LNP-formulated mRNA vaccine encoding the receptor binding domain (RBD) of the SARS-CoV-2 spike protein, induced both CD4^+^ and CD8^+^ T cell responses detectable with an ELISpot assay in a majority of trial participants (32). However, a large fraction of these responses also seems very limited in size. Additionally, BNT162b1 induced neutralizing antibodies effective against 17 spike protein variations according to a pseudovirus infection assay. Notably, in all clinically tested mRNA vaccines at least two injections were required for robust immunogenicity.

While early mRNA vaccines for rabies and influenza showed only moderate immunogenicity and did not induce cellular immune responses, recent clinical data on SARS-CoV-2 vaccines look promising. While this improvement likely reflects advances in antigen delivery, the nature of the chosen antigen also influences the quality of the induced immune response. There is need for further optimization of induction of robust cellular immunity with mRNA vaccines, study of immunogenicity in age groups not represented in phase 1 studies to date, and investigation of how single injection protocols may be achieved. Additionally, the promising saRNA technology, which has potential to overcome some of these challenges, needs to be tested in humans. Most importantly, efficacy of RNA vaccines for human protection of disease needs to be shown. The COVID-19 pandemic offers unique opportunities for this, with two phase 2/3 efficacy trials of mRNA vaccines on the way and a saRNA-based vaccine being injected into humans for the first time (43–45).

## Immunogenicity and Efficacy of RNA Vaccines in Animal Studies Looks Promising

In absence of human efficacy data, animal data needs to serve as basis for the evaluation of the properties of RNA vaccines and whether this platform will be suitable to tackle pandemics. Recent animal immunogenicity efficacy trials of both mRNA and saRNA vaccines look promising in several regards (**Table 2**).

**Table 2:** Most promising RNA vaccine animal efficacy trials against RNA viruses to date.

Target pathogen	Target molecule	Type	Species	Delivery system	Delivery route	Doses	Immunity induced	Efficacy	Control	Citation
H1N1 influenza	HA	mRNA	Mice	Protamine	i.d.	3	H+C	48/49	0/44	Petsch et al. (46)
H1N1 influenza	HA, NA, NP	mRNA	Pigs	Protamine	i.d.	1	H+C	5/5	0/5	Petsch et al. (46)
H1N1 influenza	HA	saRNA	Mice	PEI	i.m.	2	H+C	15/15	0/5	Vogel et al. (14)
H1N1 influenza	HA	taRNA	Mice	N/A	i.d.	2	H	25/25	0/5	Beissert et al. (47)
H3N2 influenza	HA	mRNA	Mice	Protamine	i.d.	3	H+C	8/8	0/8	Petsch et al. (46)
H5N1 influenza	HA	mRNA	Mice	Protamine	i.d.	3	H+C	8/8	0/8	Petsch et al. (46)
H7N9 influenza	HA	mRNA	Mice	LNP	i.m., i.d.	2	H+C	131/135	7/90	Bahl et al. (48)
SARS-CoV-2	S-2P	mRNA	Mice	LNP	i.m.	2	H+C	9/10	0/10	Corbett et al. (23)
SARS-CoV-2	S-2P	mRNA	NHPs	LNP	i.m.	2	H+C	14/16	0/8	Corbett et al. (49)
RSV	RSV-F	saRNA	Rats	LNP	i.m.	2	H+C	6/6	0/6	Geall et al. (37)
Zika	prM-E	mRNA	Mice	LNP	i.d.	1	H	19/19	1/14	Pardi et al. (50)
Zika	prM-E	mRNA	NHPs	LNP	i.d.	1	H	4/4	0/6	Pardi et al. (50)
Zika	prM-E	saRNA	Mice	LNP	i.m.	1	H	50/50	0/10	Erasmus et al. (51)
Ebola	EBOV-GP	mRNA	Guinea pigs	LNP	i.m.	2	H	5/5	0/5	Meyer et al. (52)
Ebola	EBOV-GP	saRNA	Mice	MDNP	i.m.	1-2	H+C	26/30	0/10	Chahal et al. (53)
Rabies virus	RABV-G	mRNA	Mice	Protamine	i.d.	2	H	20/20	0/5	Stitz et al. (54)
Powassan virus	prM, prE	mRNA	Mice	LNP	i.m.	1-2	H	29/29	2/30	VanBlargan et al. (55)
Nipah virus	sHeVG	mRNA	Hamster	LNP	i.m.	1	H	7/10	0/10	Lo et al. (56)
VEEV	TC-83	saRNA	Mice	CNE	i.m.	2	H	20/20	0/10	Samsa et al. (57)

Experiments with differences in challenge virus/time or dose/administration protocol were pooled, except where large discrepancies in efficacy. H, Humoral immunity; C, Cellular immunity; VEEV, Venezuelan equine encephalitis virus; PEI, polyethylenimine polyplexes; LNP, lipid nanoparticles; MDNP, modified dendrimer nanoparticle; CNE, cationic nanoemulsion; N/A, not specified.

Depending on the encoded protein, RNA vaccines are able to induce both cellular and humoral immunity in animals. For example, Vogel et al. showed that two injections of both 1.25 µg saRNA and 80 µg mRNA encoding HA induced both humoral immunity and cellular CD8 T cell responses and protected mice from lethal challenge with H1N1 influenza (14). Limitation of this and similar studies in mice is that induction of strong immune responses in these animals is a lot easier to elicit than in humans. This likely reflects species differences with regards to innate RNA sensing pathways including TLR3, TLR7, and TLR8 (58–60). Additionally, the usefulness of viral challenge efficacy studies in mice may be limited as these animals are not very susceptible to human influenza virus strains; therefore lab- or mouse-adapted viral strains are used in these experiments (61). Nevertheless, an increasing number of RNA vaccines are now reporting success in more relevant non-human primate models (49, 50).

Like all immunogenicity studies in humans, most animal efficacy studies to date have used two or three injections. However, some of the results of single-dose studies look promising (**Table 3**) (50, 53). For instance, Pardi et al. tested the efficacy of a preM-E encoding Zika virus mRNA vaccine administered with a single injection vaccine in mice and non-human primates (NHPs), which show a very similar disease phenotype and immunity to humans (50). All mice vaccinated with a single 30 µg dose and the four macaques single-dose vaccinated with 200 or 50 µg of mRNA were protected against detectable viraemia after viral challenge.

**Table 3:** Suitability of RNA vaccines for emerging pandemics.

Necessary and desirable characteristics	Do RNA vaccines fulfil this?
Safety	Yes
Tolerability	Acceptable, larger scale clinical trials needed
Efficacy	Immunogenicity promising in human studies, potent efficacy in animals – remains to be optimized and confirmed
Fast development	Yes
Fast regulatory approval	Likely yes, dependent on growing safety database
Fast and scalable manufacturing	Likely yes, dependent on approach used
Fast delivery	Likely yes, simple production facilities
Low cost	Likely yes, dependent on formulation and dose
One shot regimen	Potentially achievable based on animal data
Induction of cellular and humoral immune responses	Possible in human and animal studies, further optimization of induction of cellular responses needed
Thermostability	May be achievable, more investigation needed

While animal efficacy studies have shown reasonably promising results, translation of these findings to humans is limited with the majority of studies being conducted in small animal models. The extent to which any animal model predicts human response is much debated and highlights the importance of demonstrating proof-of concept in human studies.

## Thermostability of RNA Vaccines Remains Largely an Open Question

Thermostability of a vaccine is critical to reduce the need for a cold chain during vaccine storage and deployment, ensuring global availability of a vaccine in case of a pandemic. RNA is intrinsically unstable, being prone to hydrolysis, and needs to be stored frozen to ensure long-term stability (62). Current RNA vaccines under development for COVID-19 require -80 to -20°C storage during distribution, a significant limitation for global deployment (63, 64). Lyophilisation, freeze-drying, has previously been explored as a method to raise the thermostability of RNA vaccines (54, 65). A freeze-dried mRNA vaccine for rabies was shown to retain immunogenicity and efficacy in mice after 1-month storage at alternating temperatures of 4 and 56°C (54). Freeze-dried versions of the mRNA COVID-19 vaccines might become available eventually. Formulation of RNA with thermostable lipid nanoparticles might contribute to raising the thermostability of RNA vaccines (66). All in all, it seems likely that the thermostability of RNA vaccines will improve in the coming years.

## Discussion

RNA modification and formulation have been critical for raising the immunogenicity of RNA vaccines and will be key to further improve immunogenicity in humans. For example, lipid nanoparticles (LNPs) are one of the most promising methods for the delivery of RNA vaccines. Geall et al. showed that LNP-formulation of saRNA increased IgG titers against RSV 8-fold relative to unformulated saRNA in rats (37). Formulation with LNPs also increased induction of cytotoxic T lymphocyte levels. While the authors found that the properties of the LNP delivery system looked promising, a direct comparison of LNP formulation with other formulation methods would be informative. This reflects a general issue in the field; companies and developers are often reluctant to facilitate head-to-head comparisons. Furthermore, as the field is driven by biotech companies, experiments and even trials are not always published. The COVID-19 pandemic may contribute to resolving some of these issues temporarily. For instance, as most COVID-19 mRNA vaccines encode the spike protein, this may allow direct comparison of immunogenicity of different approaches. Additionally, the urgency of finding a COVID-19 vaccine may continue to result in faster and increased publication of clinical trial results.

Ideally, vaccines against pandemic pathogens should be protective after a single injection. Animal data on single-dose regimens looks promising for certain antigens and vaccine approaches. Additionally, to halt the early phases of an outbreak, neutralizing antibody responses may not need to be very high or durable. Hence, achievement of a single-dose regimen with an RNA vaccine might be possible. RNA modifications aimed at increasing the residual time of RNA may aid achievement of a single-dose regimen. For instance, the use of polyplex-loaded polymeric scaffolds for slow release of mRNA after injection has been explored (67, 68).

Further optimization of immunogenicity of RNA vaccines is needed. However, it will be important not to jeopardize the suitability of RNA vaccines to tackle pandemics through improving immunogenicity at the cost of increased manufacturing complexity and cost. The fact that smaller doses make production cheaper needs to be considered. saRNA vaccines look promising in this regard. As shown by Vogel et al. (14), saRNA vaccines protect mice from lethal influenza virus infection at much lower doses than conventional mRNA vaccines. However, using saRNA increases manufacturing complexity through the need for synthesis of longer RNA constructs. A recent technological advancement may help to overcome this issue. Beissert et al. developed a bipartite vector trans-amplifying RNA (taRNA) system where replicase-encoding RNA is produced in advance and is then added to the on-demand produced transreplicon of the antigen of interest. Using this system, animals were protected from influenza challenge at doses as low as 50 ng (47). Hence, taRNA promises to reduce the amount of RNA needed for immunogenicity, while promising significantly simpler production than saRNA.

The trade-off between improved immunogenicity and increased manufacturing complexity is even more pronounced for vaccine formulations. Vaccine formulations, such as the encapsulation of RNA within lipid nano-particles (LNPs) or vaccine lyophilisation, add further complexity and cost. Modeling by Kis et al. that estimated 575,000 doses of an RNA vaccine could be produced in 60h at around $0.30 per dose assumed a set cost for formulation (27). As the formulation process may make up a large fraction of manufacturing cost, different formulation approaches should be compared for associated expense, in addition to taking into account important considerations around scalability, supply chain logistics of different formulation components, and long-term stability of ready-to-use formulations.

Technological advances may also help to resolve the trade-off between manufacturing complexity and immunogenicity of RNA formulation. Indeed, the immunogenicity of saRNA molecules delivered on the interior or exterior of LNPs is similar (69). Hence, LNPs may be prepared and quality controlled in advance of an outbreak and be combined with RNA later (51).

When optimizing immunogenicity, speed of manufacturing needs to be conserved as much as possible. This needs to be considered when optimizing immunogenicity through increasing doses or vaccine formulation. Technological advances may help to overcome the trade-off between immunogenicity and manufacturing complexity.

## Conclusion

RNA vaccines fulfil many of the characteristics necessary to be useful to tackle an emerging pandemic and Disease X (**Table 3**). They can be rapidly developed and manufactured at low cost. Their safety and tolerability looks acceptable as long as disease enhancement is prevented. The big remaining hurdle is demonstrating the efficacy of RNA vaccines in humans. To this end, clinical efficacy studies are needed, which have been initiated for COVID-19 vaccines. Additionally, current clinical data suggests that cellular immunogenicity of mRNA vaccines may need to be further improved. Here, comparing and combining different RNA modification and formulation approaches may prove critical. At the same time, the cost of RNA modifications, formulations, and supply chain logistics need to be considered. The use of self-amplifying RNA vaccines and additional technological advances might contribute to raising immunogenicity without increasing manufacturing cost and complexity. The coronavirus COVID-19 pandemic is the first field test of whether RNA vaccines can be quickly developed, approved, and produced in face of a pandemic. The race for a COVID-19 vaccine may produce data that will not only answer the question of efficacy of RNA vaccines in humans, but may also advance the RNA vaccine platform to a stage where faster responses to similar future situations are possible.

## Author Contributions

JS wrote the manuscript. RS revised the manuscript. All authors contributed to the article and approved the submitted version.

## Funding

RS is funded by the Department of Health and Social Care using UK Aid funding and is managed by the Engineering and Physical Sciences Research Council (EPSRC, grant number: EP/R013764/1, note: the views expressed in this publication are those of the author (s) and not necessarily those of the Department of Health and Social Care).

## Conflict of Interest

RS is a co-founder of VacEquity Global Health, a Social Business focused on the development of a self-amplifying RNA vaccine against CoV2.

The remaining author declares that the research was conducted in the absence of any commercial or financial relationships that could be construed as a potential conflict of interest.
